# Adverse Events of the Long-Term Use of Opioids for Chronic Non-cancer Pain: A Narrative Review

**DOI:** 10.7759/cureus.51475

**Published:** 2024-01-01

**Authors:** Abdullh A Altawili, Mohammed A Altawili, Amnah H Alzarar, Noor M Abdulrahim, Haidar H Alquraish, Maryam A Alahmari, Marouj H Basyouni, Yara A Almohaya, Wafa Mohammed S Alhabshan, Abdullah Mohammed A Alshahrani, Jafar Faraj A Alamrad, Ahmad S Aljumaah, Mohammed A Alsalman, Abdullah A Alhafith

**Affiliations:** 1 Internal Medicine and Gastroenterology, King Fahad Specialist Hospital, Tabuk, SAU; 2 College of Medicine, Alaziziyah Primary Health Care Center, Tabuk, SAU; 3 College of Medicine, Najran University, Najran, SAU; 4 College of Medicine, Batterjee Medical College, Jeddah, SAU; 5 General Medicine, Dhahran Long Term Care Hospital, Dhahran, SAU; 6 College of Medicine, King Khalid University, Khamis Mushait, SAU; 7 College of Medicine, King Khalid University, Abha, SAU; 8 General Medicine, Alsnaeyah Primary Health Center, Khamis Mushait, SAU; 9 College of Medicine, King Saud University, Riyadh, SAU; 10 General Medicine, Prince Saud Ben Jalawi Hospital, Al-Ahsa, SAU; 11 College of Medicine, King Faisal University, Al-Hafouf, SAU

**Keywords:** cncp, non-cancer, chronic pain, review, long-term, opioids

## Abstract

Background: The long-term use of opioids for chronic non-cancer pain (CNCP) has drawn more attention and debate. Although opioids are frequently used to treat chronic pain, their effectiveness and safety over extended periods are still unknown.

Objectives: The purpose of this review is to provide an overview of what is currently known about the adverse events of long-term use of opioids in CNCP. It also delivers patient-centered strategies designed to mitigate these risks.

Methods: We conducted a literature search in PubMed, MEDLINE, EMBASE, and Web of Science databases. Search terms included CNCP, pain pathophysiology, opioid pharmacodynamics, opioid prescribing trends, guidelines for opioid use, and opioid side effects.

Results: Our review highlights that while opioids may provide short-term relief from CNCP, their effectiveness diminishes over time due to the development of opioid tolerance. This tolerance often leads to increased dosages, which can subsequently result in opioid dependence. Additionally, long-term opioid therapy is associated with a spectrum of adverse effects, including constipation, drowsiness, respiratory depression, and potential for drug interactions. Furthermore, our review indicates that alternative pain management strategies play a crucial role in controlling CNCP. They offer significant benefits with fewer adverse events. These strategies include non-opioid medications, physical therapy, cognitive-behavioral therapy (CBT), various interventional procedures, injection therapy, and acupuncture.

Conclusion: Using opioids to manage CNCP presents several challenges. Given these challenges, alternative treatments are being considered as viable options. Moreover, it is crucial to customize treatment plans to align with the patients' specific health requirements, existing conditions, and potential risks to ensure the best possible outcomes.

## Introduction and background

Chronic non-cancer pain (CNCP) is a pervasive and often debilitating condition, characterized by persistent pain that endures on most days for a minimum of three months and is not associated with malignant disease [1]. It affects approximately 20% of adults worldwide [2]. It is classified into nociceptive, neuropathic, and nociplastic pain, with its manifestation and impact influenced by demographic and socioeconomic factors [3,4].

The mechanism of perception of pain involves complex interactions at the level of the peripheral and central nervous systems through specialized nerve endings known as pain receptors or nociceptors [5]. When these nociceptors detect potential harm, they transmit a signal to the brain, which is perceived as pain [5]. In some individuals, the pain system can become sensitized, leading to a decreased pain threshold and an enhanced response to painful stimuli, a phenomenon known as hyperalgesia [6].

Prevalence rates of CNCP vary considerably worldwide, with some studies reporting rates as high as 33% in certain Western populations such as the United States and Western Europe [7-9]. However, rates in other regions, such as Asia and Africa, may differ due to factors like the definition of chronicity, healthcare access, and demographic factors such as age and gender [10,11]. In Africa, CNCP prevalence is between 20% and 40% [10]. Across Asia, the prevalence of CNCP in adults has been reported to vary greatly, ranging from 7% in Malaysia to 60% in Cambodia and Northern Iraq. Older adults are more likely to experience CNCP, with prevalence rates as high as 40% to 90% [11].

CNCP's effects are profound, diminishing quality of life, impeding daily activities, and limiting work productivity [12,13]. The social burden is significant, with some 20% of those affected reporting an inability to work [2]. Uncontrolled pain can lead to increased healthcare utilization and reduced productivity, creating economic strains [14,15]. Therefore, managing CNCP effectively is crucial for the well-being of individuals and societies alike. Given the complexities of CNCP and the challenges in its management, this review focuses on the adverse effects associated with long-term opioid therapy in patients with CNCP. It distinguishes between acute adverse events, which occur shortly after treatment initiation, and chronic adverse events, which develop over an extended period of opioid use. The rising physical (dose dependence) and psychological dependence on opioids for pain management, coupled with escalating safety issues, demands a comprehensive evaluation of their use to guide better clinical practices and patient outcomes [2].

## Review

Methods

We searched through four key databases: PubMed, MEDLINE, EMBASE, and Web of Science. We focused on terms associated with CNCP, including "Chronic non-cancer pain," "Pain pathophysiology," and "Pain mechanisms in CNCP." We also searched how opioids work, using the terms "Opioid pharmacodynamics", "Opioid pharmacokinetics," and "Opioid receptors." Our research delved into how often opioids are prescribed for CNCP and the nature of their use, with terms such as "Opioid prescribing trends" and "Epidemiology." We reviewed guidelines for when to use opioid therapy, using phrases of "Opioid therapy guidelines" and "Indications for opioid use." We also used terms like "CNCP assessment tools" and "Pain assessment scales”. We searched for alternatives to opioids, using terms like "non-opioid treatments for CNCP" and "Multimodal pain management." In addition to using "Opioid side effects" and "Opioid-induced hyperalgesia."

Rationale for opioid therapy in CNCP: understanding pain in CNCP

CNCP presents a complex medical challenge, categorized into various pain types that contribute to the overall discomfort experienced by patients [1]. These pain categories are nociceptive, neuropathic, and nociplastic [3]. Each has distinct pathophysiological origins and clinical presentations. Nociceptive pain occurs when peripheral nociceptors are activated in response to actual or potential tissue damage [3]. This type of pain is a protective mechanism that alerts the body to harmful stimuli [16]. There are two categories of nociceptive pain: somatic and visceral. There are two main types of nociceptive pain: deep somatic and superficial somatic [16]. Deep somatic pain, like persistent lower back pain, is dull and ill-defined, while superficial somatic pain, such as in osteoarthritis, is intense and throbbing. Visceral pain, found in conditions like irritable bowel syndrome, is characterized by poorly defined, heavy, dull sensations. Both deep somatic and visceral pain can include referred or radiating pain, making management complex [16] (Table 1).

**Table 1:** Categories of CNCP Note: references to this information are [3] and [5]. CNCP, chronic non-cancer pain; CRPS, complex regional pain syndrome; IBS, irritable bowel syndrome

Category	Type	Example	Description	Characteristic
Nociceptive pain	Deep somatic [3]	Persistent lower back pain [3]	Dull and ill-defined pain [3]	Can include referred or radiating pain, making management complex [3]
Nociceptive pain	Superficial somatic [3]	Osteoarthritis [3]	Intense and throbbing pain [3]	Aggravated by movement and weight-bearing activities. Activities such as walking, standing, or climbing stairs may worsen the pain. Resting or reducing joint movement often provides temporary relief [3]
Nociceptive pain	Visceral [3]	Irritable bowel syndrome [3]	Poorly defined, heavy, dull sensations [3]	Can include referred or radiating pain, making management complex [3]
Neuropathic	Peripheral neuropathy [5]	Diabetic neuropathy [5]	Superficial neuropathic pain: pain originating from damage or dysfunction to the peripheral nerves in the skin [5]	Burning, shooting, tingling, or numbness [3]
Neuropathic	Central neuropathic pain [5]	Phantom limb pain: pain felt in a limb that has been amputated [5]	Results from damage or dysfunction to the central nervous system [5]	Burning, shooting, tingling, or numbness [3]. Stabbing or throbbing pain, hypersensitivity to stimuli, deep aching or crushing sensations [5]
Nociplastic pain	Superficial somatic pain [5]	Allodynia (pain from non-painful stimuli) in fibromyalgia; hypersensitivity to touch or pressure in CRPS; increased pain response to temperature changes or light touch in centralized pain conditions [5]	Pain localized to or originating from the skin or superficial tissues. It can be described as a burning, tingling, or sharp sensation on the surface of the body [5]	Altered central nervous system processing and amplification of pain signals, abnormalities in pain modulation, changes in the sensitivity and responsiveness of the nerves and receptors in the skin and superficial tissues [5]
Nociplastic pain	Deep somatic pain [5]	Pain, chronic joint pain in osteoarthritis, and persistent deep pain in chronic back pain conditions [5]	Pain arises from deeper structures, such as muscles, tendons, or joints. It may manifest as aching, throbbing, or stabbing pain in the affected areas [5]	Abnormalities in central pain processing and amplification, altered neuromuscular function, sensitization of deep tissues and structures, changes in the processing of nociceptive signals within the spinal cord and brain [5]
Nociplastic pain	Visceral pain [5]	Abdominal pain and discomfort in IBS, pelvic pain in endometriosis, bladder pain in interstitial cystitis [5]	Pain originates from the internal organs, such as the gastrointestinal tract, bladder, or reproductive organs. It is often described as a deep, squeezing, or cramping pain that may be diffused or poorly localized [5].	Altered central pain processing related to the internal organs, visceral hypersensitivity, dysregulation of the autonomic nervous system, abnormal signaling and processing within the gastrointestinal, urogenital, or reproductive systems [5]

In terms of demographics, CNCP affects both sexes, but women tend to report chronic pain more frequently than men. Furthermore, certain conditions like fibromyalgia and migraines predominantly affect women [17] (Table 2).

**Table 2:** CNCP prevalence differences according to sex CNCP, chronic non-cancer pain

Demographic Group	Prevalence of CNCP	Conditions Predominantly Affecting the Group
Women	Higher reports of chronic pain	Fibromyalgia, migraines
Men	Lower reports of chronic pain	Cluster headaches, ankylosing spondylitis

In terms of disease type, CNCP is often associated with conditions such as arthritis, trauma, post-surgical pain, and various musculoskeletal disorders [17].

Opioid mechanism of action

Opioids play a significant role in modulating pain, mood, stress response, and digestion [18,19]. They can control CNCP when alternative therapeutic approaches have proven inadequate in pain management [18]. This class of drugs is not typically the first line of intervention, due to their potential risks; they are often prescribed when other treatments such as nonsteroidal anti-inflammatory drugs (NSAIDs) or physical therapy have failed to provide sufficient relief [2].

With a history spanning over 5,000 years, opioids have been employed as analgesics [20]. Substances like morphine, oxycodone, and hydrocodone bind with the opioid receptors within the central and peripheral nervous system, inducing changes in pain perception and facilitating analgesic effects [18,19].

The mu, kappa, and delta opioid receptors each have unique roles within the modulation of pain and the pharmacological effects of opioids. Mu receptors are primarily responsible for the analgesic and sedative effects of opioids and are known to mediate several adverse effects, including constipation and the risk of respiratory depression [18,19]. In contrast, kappa receptors, while also contributing to analgesia and sedation, are distinguished by their role in inducing aversive affective states and psychotomimetic effects, which encompass altered sensory perceptions and cognitive processes [18,19]. The functions of delta receptors, although not as extensively characterized, appear to contribute to pain modulation and may influence emotional regulation, offering a distinct profile of effects from mu and kappa receptors [18,19].

When these receptors are activated, they initiate a cascade of biochemical events that suppress adenylyl cyclase activity and alter gene expression [19]. They inhibit calcium channels at presynaptic sites, reducing neurotransmitter release in pain pathways and thus decreasing pain signal propagation [5,18]. In the brain's ventral tegmental area, opioids decrease GABA release, enhancing dopaminergic neuron activity and creating euphoria [19]. In post-synapse, opioids open potassium channels, leading to hyperpolarization and reduced neuronal excitability [19].

Prevalence of opioid use for CNCP

Opioid usage in CNCP management is widespread, with rates influenced by evolving clinical guidelines and policy shifts. Currently, long-term opioid use prevalence stands at 2.3%, with short-term use at 8.1%. Among patients with chronic low back pain, opioid use is at 5.8% [21]. The increasing trend in opioid prescriptions, from 0.47% in 2010 to 2.63% in 2019, demonstrates their integral role in CNCP therapy but also emphasizes the importance of prudent patient selection and monitoring [22].

Indications for opioid therapy

Opioids are indicated for a spectrum of CNCP states, including chronic back pain, severe osteoarthritis, fibromyalgia, failed back surgery syndrome, complex regional pain syndrome, chronic post-surgical pain, and migraines that severely affect life quality [23]. These conditions warrant the consideration of opioids when other treatments have not provided sufficient relief, mindful of the potential risks and benefits, informed patient consent, and structured management plans [24].

Assessment tools and parameters for CNCP diagnosis

Effective CNCP diagnosis hinges on comprehensive assessment tools that evaluate pain quality and track its progression. A thorough pain assessment includes the sensory and affective aspects, the temporal pattern, and the specific body regions affected. When possible, it should also seek to identify the pathophysiological mechanisms at play [25].

**Figure 1:**
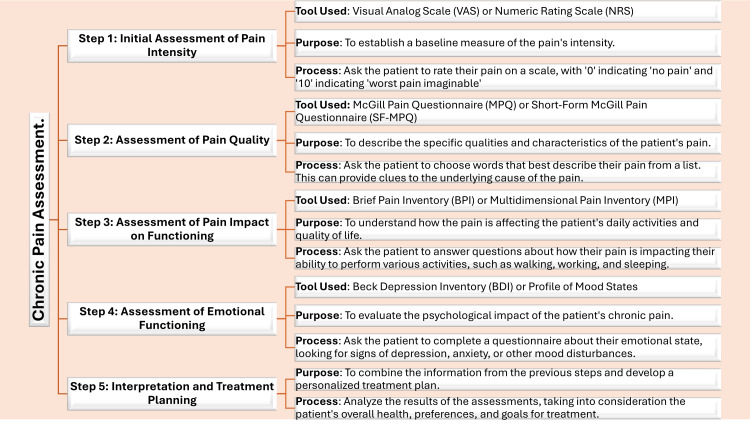
Overview of pain assessment tools The tools are based on methodologies referenced in the studies [26-30]. This image was originally provided and edited by the authors.

Pain severity is commonly gauged using scales such as the Visual Analog Scale (VAS) and Numeric Rating Scale (NRS), which allow patients to quantify their pain intensity [26]. Assessing pain quality is also essential for accurate diagnosis, with tools like the McGill Pain Questionnaire (MPQ) and the Short-Form McGill Pain Questionnaire (SF-MPQ) helping patients communicate the sensory and emotional aspects of their pain [27]. Additionally, tools like the Brief Pain Inventory (BPI) and the Multidimensional Pain Inventory (MPI) are used to assess pain duration and its impact on daily activities [28,29]. Tools like the Beck Depression Inventory (BDI) and Profile of Mood States provide valuable insights to enhance care and improve outcomes [28]. A holistic approach addressing both physical and emotional aspects fosters better pain management and overall well-being [28]. Understanding the psychological effects of chronic pain through assessments of emotional functioning is critical for devising comprehensive treatment approaches [30] (Figure 1).

Alternative management strategies for CNCP

**Figure 2:**
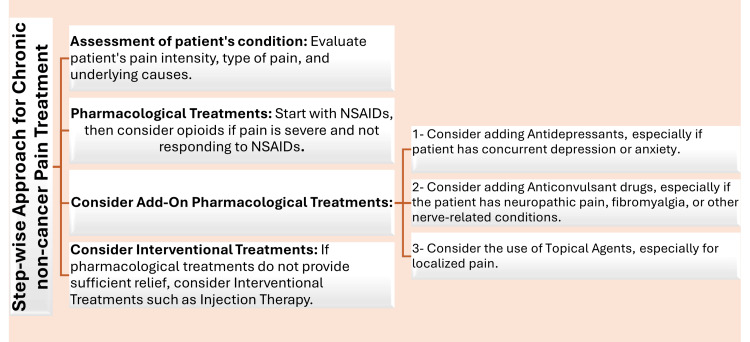
Step-wise approach of CNCP treatment by the Centers for Disease Control and Prevention This outline does not consider the specific patient's condition, preferences, or contraindications, which should always be considered when creating a treatment plan. The steps are not always linear and may need to be adjusted based on the patient's response to treatment. The aim is to provide a general idea of how interventions might progress in a typical case of CNCP. Please consult with a healthcare provider for appropriate treatment strategies. This image was originally provided and edited by the authors [2]. CNCP, chronic non-cancer pain

Both CDC clinical practice guideline for prescribing opioids for pain and guidelines for prescribing controlled substances for pain by the Medical Board of California assists doctors in managing CNCP by advising on when to prescribe pain medication, which type to choose, and the appropriate dosage to minimize addiction and overdose risks [2,24]. It emphasizes careful prescribing, low-effective doses, and vigilant patient monitoring to enhance treatment and safety [2]. In the management of CNCP, opioids are considered a viable option only when patients do not adequately respond to other lines of treatment [2,24]. Due to the inherent risks of tolerance, dependence, addiction, and adverse effects associated with opioids, healthcare providers are increasingly adopting multimodal pain management strategies that incorporate NSAIDs, antidepressants, anticonvulsants, and topical agents, allowing for a more tailored approach to each patient's needs [31] (Figure 2). This can potentially reduce the required dosage and duration of opioid therapy, thus mitigating associated risks [24,32]. Such strategies are in line with an advanced understanding of pain's complex mechanisms and the latest diagnostic advancements, representing a shift toward a more comprehensive and less opioid-dependent approach to pain relief [24,32].

Pharmacological treatments apart from opioids

Pharmacological treatments apart from opioids are shown in Table 3.

**Table 3:** Table of therapies, their classification, receptor profile/mechanism, tolerance risk, addiction profile, and adverse effects This information is from references [33] and [45]. The tools are based on methodologies referenced in studies [26-30]. This image was originally provided and edited by the authors. NSAIDs, nonsteroidal anti-inflammatory drugs

Therapies	Classification	Mechanism	Addiction	Adverse effects
Opioids	Analgesic	Opioid receptors	High	Tolerance, dependence, addiction
NSAIDs	Anti-inflammatory	Inhibit COX enzyme	Low	Gastrointestinal, renal, and cardiovascular effects
Antidepressants	Antidepressant	Affect N-methyl-D-aspartate, adenosine, sodium channels, serotonin, noradrenaline, opioids	Med-high	Cardiovascular events, potential for falls, toxicity at high doses
Anticonvulsants	Anticonvulsant	Control voltage-gated sodium or calcium channels, glutamate antagonism, augment GABA	Med-high	Fatigue, weight gain, dizziness
Topical agents	Topical analgesic	Applied to skin	Low	Local skin irritation

NSAIDs

NSAIDs are commonly utilized as the initial pharmacological intervention for CNCP due to their anti-inflammatory, analgesic, and antipyretic properties [33]. They are particularly effective for conditions like rheumatoid arthritis, low back pain, and osteoarthritis [34,35]. However, their use is not without drawbacks, as NSAIDs can lead to gastrointestinal complications, renal effects, and an increased risk of cardiovascular events [36,37].

Antidepressants

Antidepressants play a crucial role in managing CNCP, with tricyclic antidepressants (TCAs) and selective noradrenaline and serotonin reuptake inhibitors (SNRIs) [38]. TCAs inhibit the reuptake of serotonin and noradrenaline, offering relief for neuropathic pain conditions such as diabetic neuropathy and post-herpetic neuralgia, as well as other chronic pain syndromes like fibromyalgia and chronic tension-type headaches. However, their use can also lead to cardiovascular issues and an increased risk of falls, particularly among the elderly [38,39]. SNRIs, such as duloxetine, have been noted for their efficacy in treating conditions like fibromyalgia and neuropathic pain with a more favorable side effect profile compared to TCAs [40].

Anticonvulsant drugs

Anticonvulsants, including gabapentin and pregabalin, are integral to neuropathic pain management. They stabilize nerve membranes and reduce the release of excitatory neurotransmitters. These agents have been endorsed for their efficacy in treating conditions such as fibromyalgia and neuropathic pain, with common side effects including fatigue, weight gain, and dizziness [41].

Topical agents

Topical agents offer localized pain relief and are particularly useful when pain is confined to a specific area. Capsaicin and lidocaine are examples of topical treatments that have been effective in reducing pain from neuropathic conditions and post-herpetic neuralgia, respectively, with fewer systemic side effects [42-44]. Additionally, cannabinoids like cannabidiol (CBD) have emerged as potential topical analgesics, providing localized relief without psychoactive effects [45].

Interventional treatments

A comprehensive CNCP management plan integrates interventional treatments, physical and rehabilitation therapies, psychological approaches, and complementary and alternative methods (CAM) [2]. This broad spectrum of options can be customized to meet individual patient needs, aiming not only to alleviate pain but also to enhance the overall quality of life [24]. Interventional treatments can provide immediate pain relief, while physical and rehabilitation therapies work to improve long-term function. Psychological strategies empower patients to manage and cope with pain, and CAM offers additional non-pharmaceutical pain management options. Together, these modalities balance the management of CNCP, aiming to reduce the reliance on opioids and address the intricate needs of patients with persistent pain [24].

Physical, rehabilitation, and psychological approaches

Physical and rehabilitation therapies, including exercise, are essential in managing CNCP [2]. They aim to improve function and mobility, with exercise showing promise in reducing pain and enhancing function, albeit modestly [2]. Psychological interventions, particularly cognitive-behavioral therapy (CBT), focus on helping patients understand and change the relationship between their thoughts, feelings, and behaviors related to pain [46,47]. Interdisciplinary pain rehabilitation programs (IPRPs) integrate various healthcare providers' expertise, demonstrating significant pain reduction and a decrease in pain medication use [48].

CAM

CAM provide additional avenues for pain management. Spinal manipulation, massage therapy, acupuncture, transcutaneous electric nerve stimulation (TENS), and yoga are among the diverse CAM approaches [49]. While the efficacy of these treatments can vary, they offer potential benefits without the risks associated with pharmaceuticals. For instance, acupuncture has been recognized for its effectiveness in conditions like osteoarthritis and headaches [50]. Yoga, with its various forms, has shown promise for conditions such as back pain and carpal tunnel syndrome, though more research is needed to fully understand its benefits [51].

Adverse events of long-term opioid use

**Figure 3:**
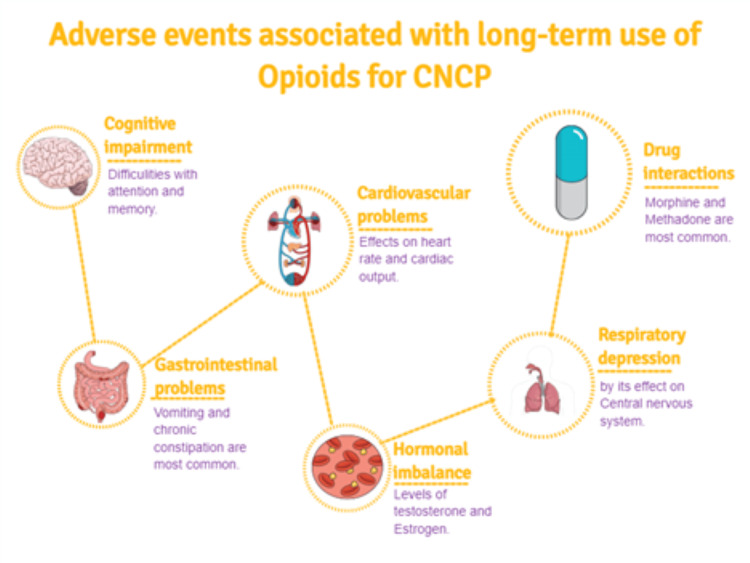
Adverse events associated with long-term use of opioids This image was originally provided and edited by the authors.

Adverse events of long-term opioid use are shown in Figure 3. When managing CNCP, measuring pain can be challenging as it often relies on the patient's description [52]. CNCP profoundly influences individuals' daily activity, with many patients indicating that it hinders their work capacity [53]. Opioids, like morphine, are often used to help with long-lasting pain in CNCP because they work by targeting specific pain receptors in the body [54]. Their main job is to make pain easier to handle by changing the way the body feels pain, making it seem less intense [55]. The use of opioids for CNCP is widely debated and researched because of safety concerns, even though prescriptions have increased [56-58].

Studies have shown that there is no clear benefit to prescribing high doses of opioids, yet the amounts prescribed have risen substantially, as seen in data from regions like Canada and England [59-61]. This is similar to what is stated in the Medical Board of California guidelines for prescribing controlled substances for pain in 2023 [24]. This increase has led to an increased risk of addiction over their effectiveness and safety. Now, it is recognized that a significant percentage of CNCP patients may develop opioid abuse or addiction [62].

Long-term opioid therapy is defined as the use of opioids on most days for greater than three months [24]. Research into the long-term benefits of opioids for CNCP suggests that any improvements in function and quality of life are typically not sustained [63]. Patients often feel more satisfied with their care in the short term, but this satisfaction does not last, indicating that opioids may not be as effective over time as once thought.

In response to these concerns, the CDC issued guidelines in 2022 for prescribing opioids for CNCP [2]. These guidelines stress that opioids should not be the first choice for treatment and that non-drug therapies and non-opioid medicines should be tried first. When opioids are used, they should be at the lowest effective dose and for the shortest time necessary, with careful monitoring for any negative side effects [2].

In the context of acute adverse effects, when initiating opioid therapy, patients may experience immediate respiratory depression, particularly when opioids are taken alongside other depressants such as alcohol or benzodiazepines [64]. This can manifest as reduced breathing rate, hypoventilation, and potentially dangerous suppression of the respiratory drive [64]. Other immediate side effects that patients often encounter include gastrointestinal issues like nausea and constipation [65].

In the context of chronic adverse effects with prolonged opioid treatment, tolerance and dependence become primary concerns [66]. Tolerance reflects the need for increasing doses to achieve the same level of pain relief, while dependence is marked by withdrawal symptoms upon cessation or reduction of the drug. These issues complicate long-term pain management and can make discontinuation of opioid therapy challenging [66]. Opioid use may also result in physical and mental side effects, such as delirium, depressive disorders, anxiety disorders, sleep disturbances, and sexual dysfunction [67]. They also interfere with the sex hormones' regular production, which could have several negative effects. Chronic opiate usage in men can lower testosterone levels, which can cause erectile dysfunction, decreased libido, and even infertility. Similar to men, women who use opioids may develop hormone abnormalities, irregular menstruation, and decreased fertility. Opioid usage during pregnancy increases the probability that an unborn child will have neonatal abstinence syndrome (NAS), a disorder marked by withdrawal symptoms [68,69]. Drug interactions pose a significant risk, especially in older adults who are more likely to be on multiple medications. For example, methadone's interactions with cytochrome P450 inducers and inhibitors can lead to ineffective pain control or dangerous side effects [70]. Morphine's interactions with other drugs can lead to serious complications like respiratory distress or serotonin syndrome [70,71].

In the context of common adverse effects, studies indicate that around 40% of patients receiving opioids report experiencing nausea, while 15% to 25% encounter vomiting [72]. Typically, nausea is a precursor to vomiting, but each can manifest independently. Patients often report that the discomfort from nausea and vomiting surpasses that of their pain [72]. These symptoms of nausea and vomiting not only reduce the quality of life but can also complicate clinical outcomes by causing electrolyte disturbances, nutritional deficiencies, and volume depletion, thereby hindering postoperative recuperation [72].

Patient-centered strategies to reduce the risk of long-term opioid Use

CDC provides guidelines for prescribing opioids for chronic pain, which recommend careful dose calculation to avoid the escalation that leads to tolerance and dependence [2]. Controlled administration and precise dosing are critical to minimize the risk of adverse events associated with opioid use [2]. Clinicians are advised to start with the lowest effective dose and to titrate slowly, monitoring the patient's response closely [2,24].

CDC recommended Prescription Drug Monitoring Programs (PDMPs). They are pivotal in the healthcare infrastructure, serving as a vigilant watchdog over opioid prescriptions and patient consumption patterns. PDMPs provide an exhaustive, accessible record of a patient's prescription history, offering invaluable insights into their opioid use trajectory. The record, which is stated by CDC in 2022 guidelines for prescribing opioids for pain, includes comprehensive details such as the prescribing physician's identity, dispensing pharmacy, prescription dates, and payment methods. By highlighting concerning patterns like high dosages or multiple provider prescriptions, PDMPs enable healthcare professionals to proactively address potential risks, suggesting alternative pain management approaches when necessary [2].

In addition, opioid stewardship programs are designed to guide healthcare providers in the responsible prescribing of opioids. These programs often include policies and procedures to reduce the risk of opioid misuse, abuse, and overdose. They emphasize the use of PDMPs to track patient prescriptions and identify potential misuse [73].

Educating patients about the risks and benefits of opioid therapy also is one of the CDC's recommendations as well as obtaining informed consent before initiating treatment. Patients should be made aware of the potential for tolerance, dependence, and other adverse effects. This education helps in setting realistic expectations and encourages patients to report any side effects promptly [2,74].

Additionally, regular follow-up appointments are necessary to assess the efficacy of the treatment and monitor for any adverse effects. Screening tools and questionnaires can be used to detect early signs of misuse or the development of mental health conditions associated with opioid use [75]. The use of urine drug testing can also help in monitoring adherence to prescribed therapies [75].

CDC also recommended the exploration of non-opioid alternatives and adjuncts for pain management. Thus, it can reduce reliance on opioids and, consequently, the risk of long-term adverse effects [2,24]. Non-pharmacological therapies such as physical therapy, acupuncture, and CBT are effective in managing chronic pain [46-51]. When pharmacological interventions are necessary, non-opioid medications, including NSAIDs and antidepressants, may be considered [33-41].

The effectiveness of these risk mitigation strategies is growing. Studies have shown that opioid stewardship programs can lead to a reduction in opioid prescribing and a lower incidence of opioid-related adverse events [73]. Patient education and regular monitoring have also been associated with improved safety profiles and better patient outcomes in the management of chronic pain [73,74].

Implementing these strategies requires a multidisciplinary approach and a commitment to an ongoing comprehensive risk mitigation plan. Thus, healthcare providers can improve the safety and effectiveness of long-term opioid therapy for patients with CNCP.

Risk vs. benefit in opioid use for CNCP

Opioids do offer short-term pain relief and may improve the quality of life for CNCP sufferers unresponsive to other therapies, yet their long-term efficacy remains uncertain and risks like tolerance and dependence are considerable [24]. Prescribing opioids for such conditions requires a meticulous assessment of the patient's pain history, treatment responses, and risk potential for opioid-related negative outcomes. The decision to prescribe opioids should be based on a thorough evaluation of the patient's pain condition, previous treatments, response to other medications, and risk factors for opioid-related harms [24].

Clinical decision-making process

This clinical decision-making process regarding the initiation, continuation, or cessation of opioid therapy in CNCP is influenced by the potential for adverse events. Clinicians must engage in shared decision-making with patients, discussing the realistic expectations of pain management, potential risks, and the importance of adherence to prescribed regimens [24].

Initiation of opioid therapy should be considered only after exhausting other therapeutic options and should be accompanied by a clear treatment goal and exit strategy [24]. Continuation of therapy should be contingent upon evidence of meaningful improvement in pain and function without significant harm to the patient [24]. Regular reassessment is crucial to determine whether the benefits of opioid therapy continue to outweigh the risks [24].

Cessation of opioid therapy may be indicated if the risks or harms outweigh the benefits if the patient experiences significant adverse effects, or if there is evidence of misuse [24]. Tapering strategies should be employed to minimize withdrawal symptoms and other potential complications associated with abrupt discontinuation [24].

In all cases, the clinician's judgment should be informed by current clinical guidelines of 2023, the patient's health history, and ongoing monitoring and assessment of the patient's response to treatment. This approach ensures that the use of opioids for CNCP is reserved for those who are most likely to benefit from it and that the risks are managed as effectively as possible.

Research recommendations

Researchers must explore the intricate issues surrounding CNCP and long-term opioid therapy. While foundational work in the field has begun, we are just starting to understand its complexities. Advancing knowledge and patient care demands a commitment to gathering high-quality, longitudinal data.

We need long-term prospective cohort studies to understand the extended impact of opioid therapy, looking past immediate pain relief to consider the possible development of opioid use disorders and the overall quality of life and functionality of patients. Randomized controlled trials (RCTs), the benchmark for treatment efficacy, must be broad in scope and designed to reflect the diverse CNCP patient population, capturing everything from pain relief to improved physical function and life satisfaction.

The varied responses of patients to opioids present a challenge that necessitates a closer examination of genetic, psychological, and environmental influences. Identifying individuals who can safely benefit from opioids will help us personalize treatments and avoid the pitfalls of a generalized approach that has complicated pain management.

Monitoring long-term opioid therapy requires developing tools that balance scrutiny with respect to patient dignity, using objective measures such as patient-reported outcomes and biological markers to assess well-being and adherence to treatment.

As opioid prescribing practices evolve due to policy and regulatory changes, it is critical to research the impact of these shifts. We need to ensure that we are adequately addressing the needs of CNCP patients without exacerbating the opioid epidemic.

Finally, we must prioritize comprehensive data on long-term outcomes to understand not only the risks but also the potential benefits of opioids for those living with persistent pain. A balanced perspective is essential to devise policies and guidelines that genuinely support CNCP patients' best interests.

## Conclusions

In conclusion, the use of opioids in the treatment of CNCP is a challenge. While opioids can provide short-term pain relief, the potential for addiction, tolerance, and adverse side effects raise questions about their long-term benefits. It is crucial to consider alternative methods such as non-opioid drugs, physical therapy, and psychological interventions as part of a comprehensive pain treatment strategy. Our proposed decision-making process optimizes the management of CNCP through comprehensive risk management and patient-centered strategies. By encouraging collaboration among healthcare systems, government health agencies, academic institutions, and clinical research bodies, we aim to enhance patient outcomes, perform evidence-based decision-making, and tailor personalized treatment strategies. When prescribing opioids, healthcare providers must carefully balance the risks and benefits to ensure patient safety and maximize long-term results. We hope that we can address these challenges effectively.
